# Complex mental health difficulties: a mixed-methods study in primary care

**DOI:** 10.3399/BJGP.2024.0818

**Published:** 2025-09-23

**Authors:** Phillip Oliver, Vyv Huddy, Ciarán McInerney, Ada Achinanya, Michelle Horspool, Kritica Dwivedi, Chris Burton

**Affiliations:** 1 School of Medicine & Population Health, Division of Population Health, University of Sheffield, Sheffield, UK; 2 Sheffield Health and Social Care NHS Foundation Trust, Sheffield, UK

**Keywords:** mental health, primary health care, mental health services, emotional regulation, personality disorders

## Abstract

**Background:**

The term ‘complex mental health difficulties’ describes long-term difficulties with emotional regulation and relationships, including personality disorders, complex trauma, and dysthymia. People with complex mental health difficulties often experience episodic and crisis-related care.

**Aim:**

To understand how general practices can better recognise people with complex mental health difficulties and provide the best care.

**Design and setting:**

A concurrent mixed-methods study was conducted with three components: two qualitative studies and a database study.

**Method:**

People with lived experience of complex mental health difficulties were consulted throughout the study. Qualitative interviews with GPs and people with complex mental health difficulties were conducted and transcripts analysed using thematic analysis. For the database element a retrospective case–control analysis was conducted using the Connected Bradford database. Integration of results was conducted using ‘following the thread’ and triangulation methods.

**Results:**

In the GP interviews, four overarching themes were identified: the challenges of complex mental health difficulties; role expectations; fragmented communication, fragmented care; and treatment in the primary care context. In the lived experience interviews, four main themes were identified: 'How I got here'; varied care experiences; traversing mental health services; and 'being seen'. In the database study element, approximately 3040 (0.3% of the database population of approximately 1.2 million) records met our criteria for complex mental health difficulties, suggesting significant undercoding. The most informative feature was the count of unique psychiatric diagnoses. From the triangulation process, five meta-themes were identified: complexity of mental health difficulties; experience of trauma; diagnosis; specialist services; and GP services.

**Conclusion:**

The current organisation of care and lack of an acceptable language for complex mental health difficulties means that patients’ needs continue to go unrecognised and 'unseen'.

## How this fits in

People who experience complex mental health difficulties are commonly seen in general practice; however, little is known about how this group are recognised or how GPs can best provide care. In this mixed-methods study, we found that GPs implicitly recognise complex mental health difficulties but lack a common language, and struggle with challenges of managing this group with limited resources and a mental healthcare system that is largely designed around the ends of the psychological morbidity spectrum. People with lived experience of complex mental health difficulties value acknowledgement of the complexity of their mental health and history of trauma, but describe their frustrations with being 'bounced around' systems. Clinical coding of mental health conditions associated with complex mental health difficulties is low, which at present limits the use of electronic health records in identifying this group.

## Introduction

Many people with mental ill health experience difficulties that are more persistent or disruptive than common mental health disorders but do not qualify as 'severe mental illness' using GP contractual criteria.^1^ Such individuals experience repeated episodes of significant anxiety and depression, with long-term unpredictable changes in mood, emotional regulation problems, and difficulties in relationships. The term 'complex mental health difficulties*'* has been proposed to describe the experiences that services need to address.^2,3^

People with complex mental health difficulties may meet the criteria for, or have been given, diagnoses such as personality disorder, complex trauma, or persistent depression (dysthymia). Comorbid substance misuse and/or neurodevelopmental issues may be present.^4,5^ In addition, this group often experience multiple socioeconomic vulnerabilities^6^ that interact with the nature of complex mental health difficulties and the design of healthcare systems, leading to care that is episodic, crisis related, and is largely delivered in general practice and emergency departments.^7–9^ Although we recognise the challenges that people with complex mental health difficulties face, we also recognise that diagnoses, particularly personality disorder, can be unhelpful and stigmatising.^3^

Complex mental health difficulties are common, with the prevalence of personality disorder alone estimated to be around 5% in the UK.^10^ However, rates of clinical coding are much lower in primary care electronic health records (EHRs). A UK study found only 1.28% of patients in a national database had a diagnostic code for personality disorder (assuming an estimated sampled population of 3.6 million).^11^ Similarly low primary care coding rates have been observed for post-traumatic stress disorders (PTSDs) and persistent depression.^12^ Such data, therefore, severely underestimate the true rates at which complex mental health difficulties are seen in primary care. For example, studies have suggested that around a quarter of patients presenting with mental health difficulties in primary care are estimated to have personality disorder.^13^

Identifying people with complex mental health difficulties and establishing how best to provide care to support people with these difficulties is important. Evidence suggests that people with complex mental health difficulties respond less well to treatments established for common mental disorders,^14–16^ and their unmet care needs are often overlooked.^17,18^ A scoping review recently conducted by our group found that the literature focusing on primary care for complex mental health difficulties was limited, with very few studies reporting relevant patient experiences.^19^ Diagnostic coding in primary care databases was scarce compared with studies that used other measures to estimate prevalence. GPs described the challenges of providing care for this group without adequate resources and specialist resources; however, there remained significant gaps in knowledge regarding how to improve the current delivery of care, which patients found to be increasingly fragmented.

We report findings from the UNSEEN study (UNderstanding SErvices for complex mENtal health difficulties). The overall aim of this study was to understand how general practices can better identify people with complex mental health difficulties and provide the best care for this group. To do this, we addressed four research questions:

- How do GPs conceptualise and recognise people with complex mental health difficulties and what are the challenges they experience when managing these patients?
- How do people with complex mental health difficulties experience care from their GPs?
- Are there features within patients’ primary care EHRs that might indicate that a patient has complex mental health difficulties?
- How do the answers to the first three questions relate to each other?

## Method

### Study design

A concurrent mixed-methods study was conducted with three components: two qualitative studies (one with GPs and one with people with complex mental health difficulties) and an analysis of a large anonymised EHR database.

### Patient and public involvement and engagement

A panel of individuals with lived experience of complex mental health difficulties was recruited from the patient and public involvement and engagement (PPIE) forums within Sheffield Health and Social Care NHS Foundation Trust and from networking with local voluntary and community sector organisations. People with lived experience of complex mental health difficulties were involved from the outset and quarterly meetings were convened that continued throughout the study to embed their perspective in the design, implementation, analysis, and dissemination of the findings. We were careful to protect the welfare of participants by offering to follow-up and de-brief after any of the meetings. The PPIE contribution to this work is highlighted throughout this article. Other contributions included suggestions for wording and language to ensure that the findings were inclusive and sensitive. One PPIE member sat on the interview panel for the research associate interviews and the PPIE group provided input on the patient-facing study documentation including the patient information sheet.

### Qualitative studies

#### GP interviews

Interviews were conducted with GPs recruited from practices based in South Yorkshire, UK through the National Institute for Health and Care Research Clinical Research Network (CRN). A topic guide, informed by the research aims and feedback from a PPIE panel set up to guide the study, was used to guide the online interviews. These were conducted, following informed consent, by an experienced qualitative researcher (the fourth author) who had no prior relationship with the research participants. Interviews took place from August 2022 to January 2023 and took 40–60 minutes to complete. Interviews were transcribed verbatim and analysed using a reflexive thematic analysis approach, following the procedure outlined by Braun and Clarke.^20^

#### Interviews with people with complex mental health difficulties

People with lived experience of complex mental health difficulties were also recruited from general practices within the CRN. Patients were identified as having complex mental health difficulties if they had a clinical code for 'personality disorder', 'complex PTSD', or 'dysthymia', and evidence of recent mental health treatment (prescription or referral). Invitations were sent by post to invite patients after a practice GP excluded individuals whose mental health was not considered stable enough at the time to take part in the study. Purposive sampling of responders was then used to strive for diversity of age, gender, and ethnic group. The PPIE panel were consulted on the interview schedule and one member took part in a pilot interview that resulted in minor changes to the ordering of the questions.

Interested participants were offered the option of telephone, face-to-face, or online video calls and given the opportunity for any necessary adjustments before the interview. All interviews were conducted by the fourth author between January and June 2023 and lasted between 60 and 120 minutes. These were recorded and transcribed for analysis as described above.

Discussion of evolving codes and themes took place among all authors in weekly team meetings. A decision was taken to halt recruitment for both GPs and participants with lived experience once the team felt that no new themes were emerging. Development of codes and themes for the patient interviews was conducted separately from the GP interviews.

### EHR study

The EHR study employed a retrospective analysis of medical records using a case–control design. The source for the data was the Connected Bradford database,^21^ which was accessed between July 2022 and November 2023. The full method and results of this work are described elsewhere.^22^ Briefly, we created a virtual cohort of adults aged between 18 and 70 years who have or might have complex mental health difficulties. This population comprised all those with a diagnostic SNOMED-CT code relating to a mental health disorder and a prescription for a psychotropic medication within the past 10 years. We excluded people with severe mental illness identified by diagnostic codes related to schizophrenia or bipolar disorder and those with dementia. Patient cases were defined by the presence of codes for suggesting complex mental health difficulties including personality disorder, PTSD, and persistent depression/dysthymia (see Supplementary Box S1 for the full criteria). Controls were all those within the virtual cohort who did not meet these criteria. We examined the association between caseness and approximately 500 000 feature-sets comprising individual codes, families of codes (through a custom taxonomy designed to represent key features of complex mental health difficulties), and combinations of codes and code families.

### Integration of results

Findings from the two qualitative studies and EHR study were brought together using the integration methods of ‘following the thread’ and triangulation.^23^ First, themes from the GP and lived experience analysis were brought together to identify common themes (or ‘threads’) and to highlight differences and tensions. Evolving themes from the qualitative analysis and scoping review were fed into the construction of the taxonomy of feature families for the EHR work. An iterative process of PPIE and stakeholder involvement allowed emerging ideas to be tested and further refined. Finally, at the interpretation stage, our findings were brought together using triangulation.

## Results

### Qualitative findings from the GP interviews

A total of 11 GP interviews were conducted. Thematic analysis of these data revealed four overarching themes:

- the challenges of complex mental health difficulties;
- role expectations;
- fragmented communication, fragmented care; and
- treatment in the primary care context.

See Supplementary Information S1, Supplementary Table S1, and Supplementary Box S2 for the GP participant characteristics and full analysis.

#### Theme 1: the challenges of complex mental health difficulties

GPs reported that complex mental health difficulties accounted for a substantial amount of their mental health (and total) workload:

*'… 50% of our consultations are to do with mental health problems. And in our practice, a lot of those are complex mental health problems. We’re dealing day in and day out with people with complex mental health problems, and I think even if we had all the resources in the world, we’d still be dealing with them, and they would still be making our consultations very difficult because often they present with physical things that are actually due to their mental health problems.'* (GP6)

They recognised the phenomenon of complex mental health difficulties in terms of the complexity of patients’ multiple mental symptoms, recent and current social problems, and their experience of being rejected by mental health services:

*'I mean, I think* […] *they are complex, they’re usually multifactorial, and there’s usually lots of different things involved like relationships, current relationships, potential childhood trauma, substance misuse, domestic abuse, poverty often, you know, perhaps being brought up in care or losing one or other parent … there’s usually … and then perhaps failure to engage with education and then all the losses that brings with perhaps not being able to get a job and so often, yeah, quite often they’re quite chaotic people, you know, they’re not living in a two up/two down married with two children and doing a nine to five job!'* (GP7)

*'Well, the main issue is that these people don’t fit within a box. And so, the main way that people present with complicated problems is when you refer them, and they are just bounced back.'* (GP8)

In contrast they did not primarily recognise complex mental health difficulties in terms of specific diagnoses. Although the term 'complex mental health difficulties' received mixed opinions, interviewees saw its potential usefulness as an umbrella term for talking with, and about, patients:

*'The other thing is about how you identify this group because they’ll be given, I mean, I personally am happy to put a Read code, a PTSD on, but again, some GPs or particularly our registrars might not feel comfortable making that diagnosis themselves, and they might think that it’s only a specialist that can do that. And for a lot of them, they’ve just got codes like depression or mixed anxiety depression, but really, what’s going on is a lot more complicated than that.'* (GP9)

#### Theme 2: role expectations

GP interviewees described perceiving unrealistic expectations of their role in managing people with complex mental health difficulties. They saw people with complex mental health difficulties as needing specialist support that GPs are neither trained nor resourced to provide and that theirs was a 'misunderstood role':

*'I have this issue of us being asked to do more and more specialist care. We’re not; you know they wouldn’t ask us to do abdominal surgery. This is specialist care ... it’s not what GPs should be doing.'* (GP6)

Although many GPs expressed concern about the role of managing patients with complex mental health difficulties, some acknowledged that with appropriate support and training they could provide effective care:

*'I would love to be able to do a little bit of psychological therapy with people, but* […] *I don’t know that much about it, to be honest, but it would be great to be able to do that.'* (GP4)

As another example of accommodating the trauma-related issues of patients with complex mental health difficulties, one GP expressed:

*'I think it helps to learn more, and I think, you know, I have tried to bear in mind the sort of trauma-informed care to realise why a person might be acting a particular way and try and diffuse the situation and … umm … ask them, you know, how they would like us to be and what can we do to help.'* (GP7)

However, as a consequence of patients being rejected by services, GPs were often in a position of providing ‘holding’ support. Although holding might have been valued in its own right (for instance, by providing continuity against a background of fractured relationships and abandonment or by helping individuals to build trust), this was rarely the case. Instead, it was viewed as a last resort (in the absence of other forms of support):

*'So, he’s become completely dependent on us, but also, there’s no other service to send him to, so I can’t get it down to less than an hour. He talks about the same thing every time, but he has to ... go through the sort of emotional rollercoaster, so it’s not therapy. I’m not doing anything, I don’t think. I’m just holding the situation because if we don’t do that, he gets very unwell, very suicidal, ringing us about a hundred times a day.'* (GP9)

#### Theme 3: fragmented communication, fragmented care

GPs described being frustrated by rejection of their referrals by specialist services: this was seen as eroding patients’ trust in the system, adding to patients’ mental health burden, and damaging the relationship between the GP and patient:

*'So quite often IAPT* [Improving Access to Psychological Therapies] *say "we can’t take this patient, they’re too complex, please refer to secondary care", we refer it to secondary care and secondary says "they don’t meet our criteria" (and we never know what the criteria are),* [so they advise referal] *back to IAPT.'* (GP7)

GPs working with diverse populations also reported a lack of culturally appropriate services for people with complex mental health difficulties:

*'And even having a shared language about mental health difficulty, I’ve no idea, you know, about how depression, low mood, how does that translate into a 70-year-old Somalian woman — I don’t know — there’s so much — I mean, it’s fascinating, but there is so much unseen, unmet needs there.'* (GP3)

The presence of newer primary care mental health services was welcomed and described by one interviewee as a *'godsend'*. However, within a short space of time these services were in some instances overwhelmed:

*'I just think it’s the reality of the unmet need which we are handling all the time, but as soon as there was a service that was there that was in any way appropriate, it quickly got overwhelmed, which I think is really, really telling of why ... what we are — the burden that we are carrying but with very limited resources and with no supervision.'* (GP3)

#### Theme 4: treatment in the primary care context

GP interviewees recognised difficulties for people with complex mental health difficulties in accessing their practice services. They acknowledged that for some patients the process of obtaining an appointment and attending a consultation can be a daunting experience. Upskilling wider practice staff about complex mental difficulties and trauma was felt to be important because of a greater risk of misunderstanding in social interactions, difficulty in understanding intentions, or problems with emotional regulation within these contexts. Promoting a non-judgemental and empathic culture for the primary care team was felt to help:

*'Recently, I think we’ve all been asked to do* […] *training in trauma-based compassion or care or something* [...] *about how people who have had childhood trauma — they’re going to interpret, you know, a simple interaction at the reception desk — they’re going to interpret that differently and then they may become hostile or defensive or because they’re experiencing what the receptionist says as a rejection and it’s taken them back there sort of clicking into their — it’s triggering how they felt as a child when they were undergoing trauma, and therefore we need to be more understanding and have a — be more tolerant of difficult behaviours with these patients.'* (GP7)

In clinical interactions, the importance of continuity of care and taking time to listen was highlighted by GPs asking about ways they have found helpful in managing complex mental health difficulties. They also explained that this understanding may not be achieved in a brief 10-minute consultation and requires slowing down and investing time over a period to build a rapport with the patient:

*'So, to get that bigger picture takes time with an individual, it takes curiosity, and it takes a willingness to slow down and to listen and to, you know, the quickest thing is to go "oh, I hear that you’re feeling sad, OK, here’s some antidepressants, here’s a leaflet for IAPT, I’ll talk to you in two weeks". That can be done in ten minutes. It will work for a subset of people who have got pure and simple depression, but it won’t work for the people with more complex mental health problems where you’ve got to try and understand what’s going on*.*'* (GP5)

The effectiveness of treatments such as antidepressants was questioned by GPs. However, they recognised that they regularly prescribed and switched medications, often without apparent benefit:

*'... you often find people, you see people* [aged] 2*1/22, and they’ve been on four different antidepressants, and none of them have worked … '* (GP5)

GPs candidly admitted that this was, in some cases, because of an internal pressure to feel that something was being done in the face of limited alternatives:

*'I think the reason that GPs prescribe antidepressants a lot of the time is because they feel like they want to do something, and they feel that it’s frustrating because of the lack of resources. It feels like what else is there left that I can do?'* (GP1)

The most positive experiences GPs reported were in the context of multidisciplinary collaborative working, both with mental health workers attached to practices or networks. The benefits of social prescribing were highlighted. However, issues with capacity of services were also highlighted:

*'I think it’s a really positive step to have mental healthcare workers attached to GP practices, and I think it’s great to have their expertise to call upon when it’s needed … '* (GP5)

### Qualitative findings from the interviews with participants with lived experience

Nineteen people with lived experience of complex mental health difficulties were interviewed. Four main themes were identified:

- 'How I got here' — acknowledging the lived experience of trauma and other health conditions as informing complex mental health difficulties;
- varied care experiences;
- traversing mental health services; and
- 'Being seen' — the search for understanding and validation.

See Supplementary Information S2, Supplementary Table S2, and Supplementary B0x S3 for the participant characteristics and full analysis.

#### Theme 1: 'How I got here'

Interviewees described the complexity of their experience of mental health difficulties. Central to this was the experience of trauma:

*'… one of my first memories is of my dad hurting my mum. A very kind of traumatic upbringing.'* (Participant [P]8)

*'I’ve always been the same since I was little, six years old when I got abused. And then I went into care from seven till I were eighteen. I got abused then, and it triggered off from there.'* (P9)

This experience appeared to influence maladaptive coping mechanisms such as self-harm and alcohol use. Interviewees talked about the impact of receiving a specific diagnosis (such as personality disorder) but this varied from positive affirmation to a negative sense of being excluded and stigmatised. The way the diagnosis was made and conveyed seemed to be important. Where there was transparency, a diagnosis was described as strengthening a sense of self and providing a focus for treatments. On the other hand, where the process was one-sided or opaque, a diagnosis felt imposed and was questioned:

*'I’d never even heard of borderline personality disorder before, you know, but when I read up on it actually, we’d been told that I’d got that, and I like read up on it, I thought blimey that’s me!* [...] *So, it sort of didn’t upset me having a diagnosis; it sort of helped because, like, it put a reason to why things are as they are sort of thing … '* (P14)

*'I were referred to SPA* [Single Point of Access] *team; then I think I had a chat with the doctor, and I had an appointment, I think it were like a 45-minute call, the next day they called me back and said that they’d diagnosed me with BPD* [Borderline Personality Disorder]*, which didn’t go down very well because how*? […] *you can’t diagnose somebody after a 45-minute telephone conversation … '* (P6)

#### Theme 2: varied care experiences

Patients described a sense of being 'passed around', referred 'into a void', or simply not being taken seriously. Some of the frustrations that patients experienced were interpreted as GPs displaying a lack of mental health awareness. However, patients with complex mental health difficulties also talked about things that they valued from their GPs. This included continuity of care and regular appointments that were seen as 'safety nets'. Patients also highlighted the importance of validation of difficult experiences and of being heard:

*'I think for me, it’s about, like, I really value being able to see the same person repeatedly, and that feels really difficult to do. Even pre-COVID, it wasn’t that easy, and now it feels impossible. So just having that consistency and not feeling like I have to start again, each time that you speak to someone. So, I’ve found that difficult.'* (P12)

*'I have a brilliant GP who knows me and understands me, and he’s a lifeline; he’s saved me so many times, more than he knows because he’s always been there and always listened and taken care of me.'* (P15)

#### Theme 3: traversing mental health services

Secondary care services were viewed as being inaccessible with long waiting lists making patients feel that they were essentially 'lost in the system'. Patients raised concerns in particular around the response of services at times of crisis, describing difficulties in accessing help, lack of coordinated care, being left alone, a sense of rejection, and a lack of follow-up care:

*'… About two years ago, I was alone in my house with my one-and-a-half-year-old child. I was suicidal, I had self-harmed, and I rang the crisis team, and again, I had to leave a voicemail; they rang me back fourteen hours later that time. And had no advice for me beyond, "Oh, you left us a voicemail. Is everything OK?", "Well yeah, I’m alive, and my daughter’s alive". And that was it, again, no follow-up, no nothing, just OK, well we’ve checked in, she didn’t actually kill herself, her daughter’s alive, so that’s case closed*.*'* (P16)

#### Theme 4: ‘Being seen’

Some participants felt that their mental health practitioners did not fully see or understand them as individuals; instead, focusing solely on their diagnosis or symptoms fitting into a predefined framework:

*'I’ve always felt as though they want to put me in a box, like whether that’s about diagnosis or treatment or whatever, just they want to put me in a certain box, and then they know what to do with that, they know the pathway that that person then takes. But I’ve never quite fitted in any of the boxes. So, it’s almost like they haven’t quite known what to do with me*.*'* (P12)

People with lived experience of community mental health teams emphasised the need to be heard, believed, and valued in their interactions with mental health services. This need for validation and understanding was particularly important for participants who felt that although they were ‘screaming to be heard’ and understood, their experiences and distress were not acknowledged or validated by mental health professionals. One participant jokingly spoke about the research study and the acronym ‘UNSEEN’ stating:

*'… I like the way it’s called ‘UNSEEN’, because you don’t get seen, you don’t … ' (P5)*

*'I haven’t seen anyone for five years, and I’m literally just waiting on a list to see someone sometime when somebody decides to get to my name, and I’m not even sure who that might be … you feel ignored by the powers that be … '* (P13)

Another participant described how the media’s portrayal and propagation of mental health awareness often fell short of addressing their needs:

*'And all you hear all the time are these stars saying "oh it’s good to talk, oh it’s good to … " — it’s good to talk if there’s somebody there to listen! You know, it’s alright saying it’s good to talk — who are you going to talk to?'* (P13)

### EHR findings

The study population comprised approximately 155 470 individuals with a mental health diagnostic code and prescription in the preceding 10 years identified from the Connected Bradford database (13.7% of the total population of approximately 1.2 million). Of these, approximately 3040 (2.6% of the study population and 0.3% of the total database population of approximately 1.2 million adults) met our criteria for caseness, which is significantly lower than expected from epidemiological data. Clinical coding of many features, such as those relating to trauma, were also sparser than would be expected. Only one feature was found with any significant discriminative value: the count of unique psychiatric disorders. However, we identified several additional features that could have potential value in a less sparse dataset. These included binary features (for example, presence or absence of prescription for antipsychotic medication), continuous features (for example, entropy of non-attendance), and counts of features (for example, concerning behaviours such as self-harm and substance misuse).^22^

### Triangulation of findings

The findings from the two qualitative studies and the EHR study were drawn together through a process of triangulation to produce a joint display of integrated findings. Through team discussions potential common overarching themes were explored visually and narratively. Five meta-themes were identified from triangulation of the results from the separate studies:

- complexity of mental health difficulties;
- experience of trauma;
- diagnosis;
- specialist services; and
- GP services.

**Figure 1.**
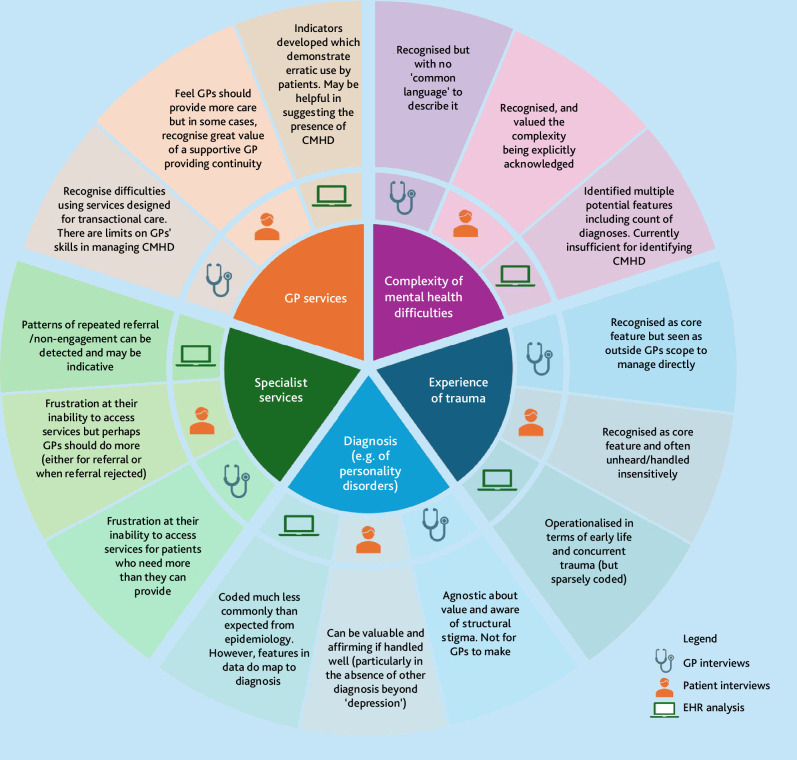
Summary of triangulation of findings. CMHD = complex mental health difficulties. EHR = electronic health record.

These are summarised in Supplementary Box S4 and Figure 1.

Figure 1 depicts the five meta-themes (central sections) that emerged from triangulating data from the two qualitative studies and the EHR study findings with illustrative descriptions (outer sections) for each of the contributing data sources. For example, the meta-theme ‘Experience of trauma’ was considered a core feature of both GP and lived experience interviews but although possible to derive features in EHRs related to trauma experiences, these were infrequently coded.

## Discussion

### Summary

Complex mental health difficulties were recognised by both GPs and people with lived experience who valued the complexity of their mental health problems being explicitly stated. Despite this, there lacked a common language between GPs and patients. GPs were themselves agnostic about the value of a diagnosis of personality disorder, recognising the structural stigma associated with this. But although concerns about stigma were echoed by patients, this was countered by the validation that a diagnosis of 'surely something more than depression' can bring. Experience of trauma appears to be a core feature of complex mental health difficulties and although GPs were acutely aware of being left as the sole resource for patients with such complex needs, they reported being overburdened and lacking the confidence and resources to provide ongoing, stable relationship-based support that can be valuable to many patients. At present, EHRs are unlikely to be a useful tool alone for identification of people with complex mental health difficulties because of sparse coding of diagnoses and potentially useful features.

### Strength and limitations

A key strength of our study was the mixed-methods approach, which allowed triangulation of GP and patient experiences to provide greater depth and validation to our findings and a wide perspective on the current unmet needs in primary care. This is the first study, to our knowledge, that has taken this three-pronged approach to complex mental health difficulties. Our use of the term 'complex mental health difficulties' was influenced by the MIND consensus statement^3^ and local patient and public involvement, and was well accepted by our patient participants. However, most participants in the patient interviews were diagnosed with personality disorder, and other complex mental health problems may have been less represented in the data.

There are some limitations to the transferability of the current findings. Despite our attempts to recruit participants from practices from areas with underserved populations, the majority of the participants with lived experience were white British. Given that GPs reported a lack of culturally appropriate services, the opportunity to explore this from the perspective of people from minoritised groups was limited. There was also an underrepresentation of males in both qualitative studies and a predominance of experienced (>10 years) GPs.

The findings from the anonymised database study were limited by the lack of diagnostic coding in the patient records. Given the expected prevalence of 3%–5% in the general population^10^ and 20%–25% of primary care attenders^13^ with any mental health difficulty, the 0.3% prevalence in our database population represents approximately tenfold underdiagnosis (or underrecording of diagnoses). Despite these limitations, we identified several features that appear promising.^22^

Future research should be conducted to explore potentially promising EHR features and to expand on the qualitative findings by addressing the limitations described above. Mixed-method approaches using EHR and qualitative data could also be used to explore other complex clinical presentations.

### Comparison with existing literature

Previous research has indicated that GPs report relying on a ‘gut feeling’ when recognising people with complex mental health difficulties.^9^ Examining the wider literature, this clinical instinct can be seen to comprise multiple aspects including psychosocial, diagnostic, and healthcare utilisation complexities,^19^ themes that were echoed in the present study. General practice is recognised as having an important role in the management of those with complex mental health difficulties.^9,24^ However, the challenges faced by GPs in our interviews, such as being left with the sole responsibility of providing care, emotionally overwhelming work under significant workload pressures, and lack of resources or specialist support, is widely reported.^9,24–29^ That these challenges continue despite changes to UK mental health services, such as those outlined in the NHS Mental Health Implementation Plan 2019/20–2023/24,^30^ is disappointing and suggest that this area of need remains unmet.

Given that so much care takes place within general practice, understanding the experiences and views of those with complex mental health difficulties is essential. It is known that people with lived experience of complex mental health difficulties report negative experiences navigating the wider healthcare system;^31^ however, much less is known about experiences in general practice. A systematic scoping review conducted by the authors in preparation for the UNSEEN study found few previous relevant studies.^19^ Thematic analysis conducted on the limited number of qualitative studies identified in this scoping review found three main themes: the recognition of complex mental health difficulties in primary care; the work of caring for people with complex mental health difficulties; and patient priorities. One qualitative study on prescribing for personality disorder found that patients valued making a connection with their GP and feeling listened too but that they also reported experiencing feeling rushed, dismissed, and uncared for in some cases.^32^ Participants with lived experience in our study also reported mixed experiences with their GPs. Positive experiences included continuity of care and feeling validated, while the sense of being 'passed around' or 'lost in the system' were frequent sources of frustration. Patients also highlighted the importance of acknowledging and addressing their past experiences, which reflects the increasing recognition of trauma-informed care in managing complex mental health difficulties.^33^ From the wider literature, the positive experience of an ongoing stable relationship with a GP was described in a qualitative study specifically looking at ‘holding’ relationships in long-term conditions.^34^ In that study, GPs explicitly acknowledged the value of holding both for their patients and themselves, which contrasts with our findings where GPs spoke mostly of their frustrations and challenges in holding this group of patients.

Our attempts to identify features from general practice EHRs that could indicate complex mental health difficulties were hindered by low rates of clinical coding. Based on epidemiological studies we would have expected prevalences approximately 10 times higher than those observed. Underrepresentation of diagnostic coding for complex mental health difficulties in EHRs compared with expected prevalence has been reported in other primary care database studies,^35–37^ suggesting a poor recognition of complex mental health difficulties in general practice.

### Implications for research and practice

Although people with complex mental health difficulties commonly fall between NHS services for uncomplicated common mental disorders and those for severe mental illness, the development of services linked to the NHS Community Mental Health Framework^38^ and the emergence of new services commissioned through primary care networks provide an ongoing opportunity to improve care for those with complex mental health difficulties. However, it is imperative that GP and patient perspectives on complex mental health difficulties are kept central to the transformation of mental health services in the UK to ensure that new services adequately engage with and cater to the unmet needs of this important group of patients.

A recent task force recommended a role for extended primary care teams in caring for those with complex mental health difficulties.^39^ The emerging emphasis on improved relational continuity in primary care^40^ also provides an opportunity to promote the building of therapeutic relationships that patients value. Ultimately, however, services need to recognise the importance of this problem and find ways to maximise and demonstrate the value of supportive primary care.

The results from the present study have been used to create a guide for GPs that contains recommendations for working with people with complex mental health difficulties. These recommendations were written following a stakeholder discussion event in September 2023 and reviewed by our PPIE group. We hope that this guide represents a first step to making complex mental health difficulties more visible and to help better support patients with complex mental health difficulties in primary care.^41^ A summary of the recommendations from this guide are shown in Box 1.

Box 1. **Suggestions to help GPs in managing patients with complex mental health difficulties**

**What can you do?**

- **Listen, validate, explain, and support**Many patients will not have received a diagnosis beyond depression or anxiety. Explain that you recognise that their mental health difficulties and experiences extend beyond this.
- **Consultations**Consultations with people with complex mental health difficulties take time. Plan for this where possible, consider flagging this on patient records so that staff are aware. Some people prefer in-person appointments, others prefer the separation that a telephone call allows.

- **Continuity of care**Continuity helps patients build trust and learn that they have been heard. This is important because many people with complex mental health difficulties find it difficult to trust others and know if they have been heard. It does not have to be for every contact.

- **Focus on life situations**Consultations may be in response to crisis or a deterioration in mental health, often precipitated by social difficulties. Acknowledge these with empathy. While it is valuable to know about available support, signposting alone may not address the emotional needs that are present in a crisis.

- **Set expectations when referring**Be prepared to have open discussions when referring, especially where long waiting times are expected or at the margins of referral thresholds.

- **Protect yourself**Patients with complex mental health difficulties may be in distress, express suicidal thoughts, and describe trauma explicitly. The psychological burden of this on you can be significant. It is important to find ways in which your practice team provides a supportive environment to discuss or debrief, and to ensure that no person’s workload burden is too heavily weighted towards complex mental health.

In conclusion, complex mental health disorders pose particular challenges for patients and for general practice. The current resources for and organisation of care limits GPs' ability to deliver the necessary continuity of care and trust for patients to benefit. The lack of an acceptable language or terminology for complex mental health difficulties means that patients’ needs continue to go unrecognised and 'unseen'.

## Open access

This article is Open Access: CC BY 4.0 licence (http://creativecommons.org/licences/by/4.0/).

## Data

The participants of the qualitative study did not give written consent for their data to be shared publicly, and due to the sensitive nature of the research supporting data is not available. The Connected Bradford Research Database is hosted by Bradford Teaching Hospitals NHS Foundation Trust and cannot be shared publicly. Data can only be made accessible to researchers on completion of a data access application and is submitted to the Connected Bradford Governance Board for scientific review. For further information on the data access process, please contact Connected Bradford on cBradford@bthft.nhs.uk or via the following website: https://www.bradfordresearch.nhs.uk/our-research-teams/connected-bradford
